# Overcoming roadblocks in the development of vaccines for leishmaniasis

**DOI:** 10.1080/14760584.2021.1990043

**Published:** 2021-11-02

**Authors:** Paul M. Kaye, Sakshi Mohan, Carsten Mantel, Melissa Malhame, Paul Revill, Epke Le Rutte, Vivak Parkash, Alison M. Layton, Charles J.N. Lacey, Stefano Malvolti

**Affiliations:** a York Biomedical Research Institute, Hull York Medical School, University of York, Heslington, York, UK; b Centre for Health Economics, University of York, Heslington, York, UK; c MMGH Consulting, Zurich, Switzerland; d Department of Epidemiology and Public Health, Swiss Tropical and Public Health Institute, Basel, Switzerland; e Department of Public Health, Erasmus MC, University Medical Center Rotterdam, Rotterdam, The Netherlands

**Keywords:** Leishmaniasis, vaccines development, experimental models, disease modeling, health economics

## Abstract

**Introduction:**

The leishmaniases represent a group of parasitic diseases caused by infection with one of several species of *Leishmania* parasites. Disease presentation varies because of differences in parasite and host genetics and may be influenced by additional factors such as host nutritional status or co-infection. Studies in experimental models of *Leishmania* infection, vaccination of companion animals and human epidemiological data suggest that many forms of leishmaniasis could be prevented by vaccination, but no vaccines are currently available for human use.

**Areas covered:**

We describe some of the existing roadblocks to the development and implementation of an effective leishmaniasis vaccine, based on a review of recent literature found on PubMed, BioRxiv and MedRxiv. In addition to discussing scientific unknowns that hinder vaccine candidate identification and selection, we explore gaps in knowledge regarding the commercial and public health value propositions underpinning vaccine development and provide a route map for future research and advocacy.

**Expert opinion:**

Despite significant progress, leishmaniasis vaccine development remains hindered by significant gaps in understanding that span the vaccine development pipeline. Increased coordination and adoption of a more holistic view to vaccine development will be required to ensure more rapid progress in the years ahead.

## 1. Introduction

Leishmaniasis is endemic in 95 countries worldwide and the cause of significant morbidity and mortality. The notion that prevention of leishmaniasis can be achieved through vaccination is supported by a significant body of experimental and epidemiological data, but currently no vaccine exists for human use. This review addressed some of the major impediments to the development of vaccines for human leishmaniasis.

### 1.1. Leishmaniasis: the clinical and public health context

The leishmaniases are a collection of globally important neglected diseases caused by several species of the protozoan parasite *Leishmania*. It is estimated that up to 1 billion people are at risk of infection [1,2]. Based on the most recent data, between 498,000 and 862,000 new cases of all forms of leishmaniasis occur each year resulting in up to 18,700 deaths and 1.6 million disability adjusted life years (DALYs) lost [3]. Transmitted by the bite of phlebotomine sand flies, the leishmaniases disproportionately affect populations in low- and middle-income countries (LMICs). In addition to the obvious effect on health during clinical disease, there is a growing appreciation of the impact of long-term sequelae associated with different forms of leishmaniasis, notably on mental health [4]. For example, the DALY burden associated with cutaneous leishmaniasis was estimated to be up to seven-fold higher when accounting for major depressive disorder [5]. Similarly, leishmaniasis may have major impacts on economic prosperity at the individual and community level, through reducing an infected individual’s ability to work and the caregiving requirements that fall on wider families and communities [6]. In one study in Sudan, 75% of households were reported to incur catastrophic out-of-pocket costs amounting to up to 40% of annual income when a family member required treatment for visceral leishmaniasis [7].

Underpinning the geographic distribution and varied clinical presentation of the leishmaniases is a complex evolutionary relationship between vector, parasite and host [8]. At least 19 species of sand fly are capable of supporting the development of *Leishmania* and have been incriminated as vectors, and human leishmaniasis has been attributed to at least 20 species of parasite belonging to two sub genera, *Leishmania* (*Leishmania*) and *Leishmania* (*Viannia*) [8]. Human genetics almost certainly plays a role in determining disease outcome, though well-evidenced examples are few and far between [9]. Other factors such as malnutrition [10], co-infection [11] and socio-economic status [12] also contribute, with leishmaniasis described as reflecting a vicious cycle of poverty and infection [13].

Clinically, the leishmaniases may be loosely sub-divided into tegumentary leishmaniasis (affecting the skin and mucosae) and visceral leishmaniasis, involving the systemic organ systems. With an annual reported incidence of over 600,000 new cases across 55 countries, the tegumentary leishmaniases represent the greatest disease burden. These include localized cutaneous leishmaniasis (LCL), disseminated cutaneous leishmaniasis (DL), diffuse (anergic) cutaneous leishmaniasis (DCL), mucocutaneous leishmaniasis (MCL) and post-kala-azar dermal leishmaniasis (PKDL). Each is typically but not uniquely associated with specific parasite species. For example, *L. major* and *L. tropica* are responsible for most LCL in Africa, Asia, and the Middle East, whereas LCL in the Americas is associated with *L. mexicana, L. peruviana, L (V.) guyanensis* and *L. (V.) braziliensis*, among others. Associations between parasite species and disease presentation are however not prescriptive. In Ethiopia, the full spectrum of tegumentary disease is associated with infection by *L. ethiopica* and inter-species hybrids are being discovered in many regions of the world [14,15], further complicating this picture.

Brazil, East Africa and South Asia carry the burden of visceral leishmaniasis, a disease that is invariably fatal in the absence of treatment and responsible for most *Leishmania*-attributable deaths [2]. PKDL is a chronic stigmatizing skin condition that develops in 5–30% of patients successfully treated for VL and affects quality of life particularly in young adults and females [16]. PKDL patients also provide a reservoir for *Leishmania* transmission and represent a significant risk to VL elimination programs [17,18].

### 1.2. Leishmaniasis – the life cycle and immune control of infection

Infection with *Leishmania* parasites is initiated during sand fly bite, with regurgitation of metacyclic promastigotes into the host dermis. From a holistic standpoint, transmission involves not only the transfer of parasites, but also reflects the biological properties of sand fly-derived proteins, parasite-excreted phosphoglycans and components of the sand fly microbiota [19]. Collectively, this microenvironment is permissible to infection of a variety of host cells including neutrophils, monocytes, tissue resident dermal macrophages and stromal cells. Whilst conversion to intracellular amastigotes may occur in many cells types, replication of amastigotes is more commonly associated with parasitism of mononuclear phagocytes where it occurs within a prescribed parasitophorous vacuole/phagolysosome [20]. This intracellular lifestyle largely dictates the nature and efficacy of the ensuing acquired immune response. Whilst B cells are activated and can produce copious quantities of antibodies that have utility in diagnosis, these are thought to be ineffective at killing intracellular parasites. A role for antibodies in limiting cell to cell transfer and parasite dissemination has not however been formally disproved. Antibodies may also facilitate killing of infected cells through antibody-dependent cellular cytotoxicity (ADCC), though again this has not been formally demonstrated. In contrast, several decades of research in experimental models and in patients has firmly established the role of T cell mediated immunity in determining the outcome of natural infection and as the primary mediator of vaccine-induced protection, at least in animal models. Tcell-derived cytokines (notably interferon-γ; IFNγ) serve to enhance the innate leishmanicidal properties of macrophages and thus promote cure, whereas disease progression is associated with cytokines that either directly inhibit macrophage leishmanicidal activity or skew T-cell differentiation away from IFNγ production. Regulatory cytokines, notably interleukin (IL)-10 produced by T cells and other cells including macrophages and B cells, play an important role in fine-tuning these responses and maintaining a balance between immunity and immunopathology. Cytotoxicity focused on infected macrophages whose function has been depressed by intracellular parasitism may allow phagocytosis by cells with greater leishmanicidal activity, providing an alternate host protective mechanism. Although both CD4^+^ and CD8^+^ T cells play a role in these host protective pathways, both can also contribute to pathology and most aspects of clinical disease are immunopathologic in nature. The immunology and immunopathology of leishmaniasis are reviewed in detail elsewhere [21–23].

### 1.3. Vaccines for leishmaniasis – current state of the art

There have been many recent reviews of leishmaniasis vaccine development [21,24–30] and only a summary is warranted here. First-generation vaccines, comprising whole killed *Leishmania* promastigotes, usually adjuvanted with *M. bovis* BCG and/or Alum, had been shown to have some therapeutic promise, but have not been developed further. However, prophylactic vaccine trials in humans have proved disappointing, with a recent meta-analysis of clinical data finding evidence of immunogenicity but not protection [27]. A new generation of live genetically attenuated (GA) vaccines for leishmaniasis have now been developed, showing great promise in experimental models [31], including cross protection by a *L. major* centrin^−/−^ vaccine against vector-transmitted *L. donovani* infection in hamsters [32]. These are discussed more fully elsewhere [33]. Second-generation subunit vaccines (including peptides and proteins in a variety of adjuvant and delivery systems [34];) have shown promise in various experimental models of cutaneous and visceral leishmaniasis. Complete protection (e.g. failure of lesion development or lack of visceralisation) has rarely been demonstrated, however, and few such candidates have progressed to human clinical trial. Notable in this regard are various incarnations of a poly-protein fusion adjuvanted with an oil emulsion developed by the Infectious Diseases Research Institute [35]. Subunit vaccines for canine VL have shown sufficient evidence of protection to warrant licensure [36], but their formulations are unsuitable for human use. Third generation, or DNA-based vaccines have been shown to be effective in rodent and simian models [37] and one, the adenovirus-based vaccine ChAd63-KH, has been shown to be safe and immunogenic in healthy UK volunteers [38] and in Sudanese patients with persistent PKDL [39]. The results of a randomized, placebo-controlled efficacy trial to assess the therapeutic benefit of vaccination with ChAd63-KH in PKDL patients are expected in 2022. mRNA vaccines, which have risen to the fore as a result of the COVID-19 pandemic [40], have yet to be fully explored in the context of leishmaniasis, with only in vitro studies on candidate antigen expression reported to date [41]. Nevertheless, as for vaccine development for malaria and other NTDs [42,43], adoption of this technology within leishmaniasis vaccine development programs is likely to be rapid.

## 2. Roadblocks along the path to vaccine development

### 2.1. The breadth of the challenge

**Figure 1.**
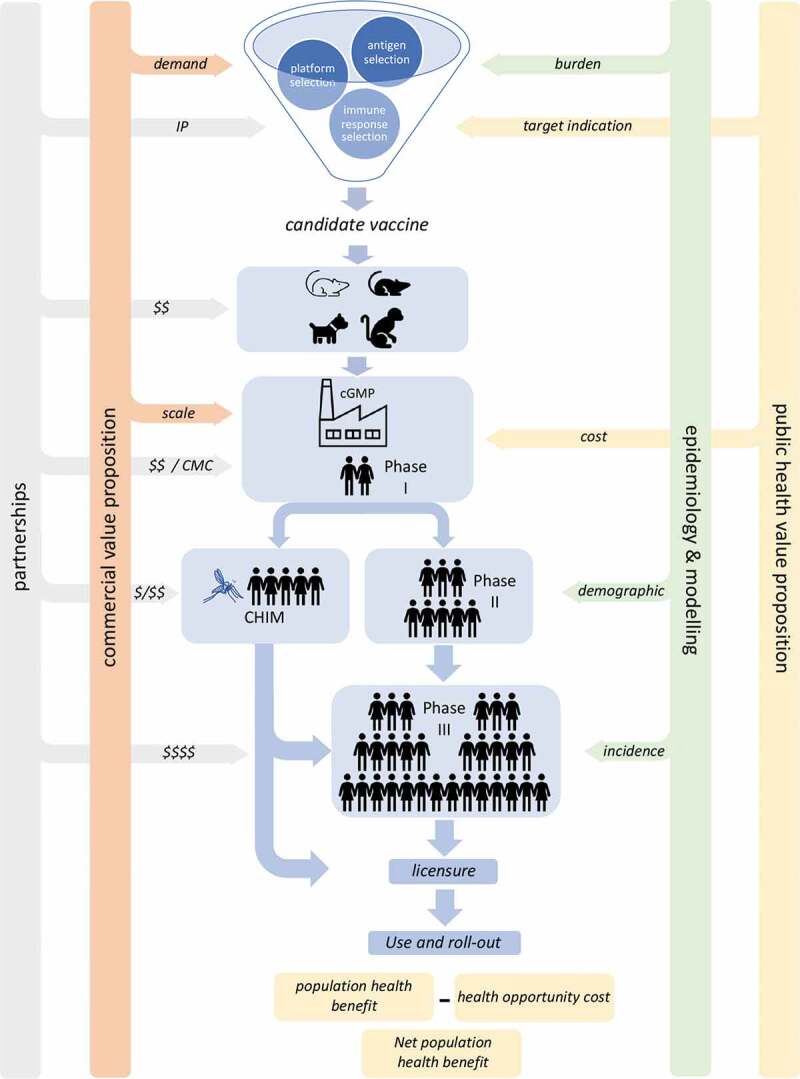
Factors influencing the development of leishmaniasis vaccines

Developing any new vaccine is a complex, multistep process fraught with scientific and practical challenges many of which are amplified in the context of vaccines for neglected diseases [44,45]. These challenges or roadblocks may be evident at various stages along the conventional linear ‘laboratory to clinic’ development process, but also arise from knowledge gaps that negatively impact on rational vaccine design, manufacture, deployment, and perception of public health value (Figure 1). Here, we focus on identifying key roadblocks to leishmaniasis vaccine development across this broad spectrum of inter-disciplinary activities. We have taken a disease- and vaccine-agnostic approach to the discussion, as many of the principles apply equally to vaccines targeting VL and CL and in either a prophylactic or a therapeutic setting. Where there are specific considerations, these have been noted.

The central column depicts the linear development process from discovery science through preclinical models, manufacturing and into human trials. Not depicted for clarity is the significant level of iteration that may occur within the processes depicted in the ‘funnel’ and through preclinical models. Side bars indicate where additional insights are required to develop a robust case for progression of a vaccine through to licensure. These may be vaccine candidate agnostic or require insights specific to each candidate. The roadblocks identified at each stage are discussed further in the text. CHIM, controlled human infection model; cGMP, current Good Manufacturing Process; CMC, chemistry, manufacturing, and controls; IP, intellectual property; $, investment. (Original Figure)

### 2.2. Candidate antigen selection

Typically, candidate vaccine antigens are identified by: i) targeted antigen discovery using immune cells or serum to directly identify antigens recognized following parasite exposure [24,30], ii) reverse vaccinology, employing bioinformatic analysis of *Leishmania* proteome or genome data [46–48], iii) reverse vaccinology based on an understanding of human genetic control of leishmaniasis and peptide elution from HLA molecules [49], iv) identifying sand fly salivary components that facilitate infection [50,51], and v) serendipity, arising as an offshoot of fundamental research on parasite biology and/or the host–pathogen interaction. The latter reflects a major knowledge gap in the foundational step of vaccine antigen selection, namely that we have few if any bona fide virulence factors identified in *Leishmania*. Best characterized are the surface lipophosphoglycan (a challenging glycoconjugate for vaccine development [52]), the protease gp63 [53] and a variety of components of the parasite secreted via exosomes [54]. As if by design, *Leishmania* does not possess a single ligand responsible for infection of phagocytes, lacks secretory organelles delivering invasion-related proteins, does not deliver virulence factors via dedicated pathways and does not have a single ‘toxin’ responsible for disease. Thus, key elements associated with successful vaccine development for other pathogens are absent. Future exploration of the abundant hypothetical proteins encoded in the *Leishmania* genome may help identify mechanisms of virulence more directly targetable by vaccines and a paradigm shift in how vaccine antigens are selected. An alternative explanation for the plethora of candidate antigens showing success in animal models may be that once triggered, even by limited antigen release from a few dead parasites, vaccine-induced protection is then mediated relatively nonspecifically, reliant on promiscuous killing of parasites by activated macrophages. Generation of parasites deficient in select vaccine antigens by CRISPR/cas9 engineering provides a route to test this hypothesis. Novel methods for epitope selection based on high throughput T cell receptor sequencing are now available [55] and will likely provide an even more granular view of human antigen recognition across the leishmaniasis disease spectrum and after cure. Whilst small-scale pilot studies will provide initial insights, appropriately designed epidemiological studies will be required to link such data to disease outcome and control for confounding factors affecting immune recognition. As parasite immune evasion strategies have been honed by their evolution in response to naturally induced immune responses, due consideration should also be given to identifying novel pathways of immunity that can be induced by vaccination without an over-reliance on vaccines trying to mimic natural immunity.

Given the background of so many vaccination studies in animals and with so many candidate antigens (albeit many selected on relatively weak evidence), it is pertinent to ask whether more are needed. Do we already have a sufficient armory of candidate antigens and what is lacking is the capacity to evaluate these across different platforms and in models that are sufficiently predictive of human response, or indeed in humans themselves? These types of studies (involving harmonized protocols and end points, potentially complex models of vector transmission, IP discussions related to mixing of platform technologies and the like) are challenging for any single organization to conduct, particularly if their core business is discovery science. Or do we have to re-set and apply more rigorous selection criteria for candidate antigens based on large-scale interrogation of human immune responses. Either way, the development of a comprehensive framework for identifying and evaluating existing and emerging candidate antigens may facilitate progress in this regard, e.g. following the model of CEPI (https://cepi.net/get_involved/cfps) and/or the Solidarity study for COVID-19 treatments (https://www.who.int/emergencies/diseases/novel-coronavirus-2019/global-research-on-novel-coronavirus-2019-ncov/solidarity-clinical-trial-for-covid-19-treatments). Such a framework could also incorporate evidence of target disease burden and vaccine demand as selection criteria given that, as discussed below, these factors will ultimately determine the public health and commercial viability of a vaccine.

### 2.3. Exploiting parasite genomics to further identify candidate antigens and mechanisms of protection

The *Leishmania* genome contains approximately 8500 genes, many encoding ‘hypothetical’ proteins of currently unknown function. Genome instability and aneuploidy, together with a sexual stage in the sand fly vector that facilitates the generation of ‘genetic hybrids’ [56,57] all serve to complicate *Leishmania* genetics. To date, our increased knowledge concerning genetic diversity in *Leishmania* has not been used to directly identify candidate vaccine antigens or novel mechanisms of immunity. The recently reported ability to generate hybrids in vitro may allow use of forward genetics to address these challenges [58]. As a result of polycistronic transcription, mRNA abundance in *Leishmania* is controlled by gene dosage [59] and post-transcriptionally and/or post-translationally [60]. Transcriptomic studies have identified stage-specific differences in mRNA abundance, providing opportunities for reverse genetic approaches to identify candidate antigens associated with infective stages of the life cycle, e.g. through high throughput DNA vaccine screening [61]. The development of high throughput protein expression systems may also facilitate more rapid identification of candidate vaccine antigens [62]. Recently, application of microbial genome-wide association studies (mGWAS) led to the identification of genetic markers associated with miltefosine resistance in *Leishmania infantum* [63] and similar approaches could be used to identify potential *Leishmania* vaccine candidate antigens, as mooted for other pathogens [64]. The full power of such approaches is only likely to be realized through application of knowledge on the epidemiology of disease in different geographic settings. This will require insights into parasite epidemiology and transmission, information that will be equally valuable in the design of future clinical trials. The recent application of CRISPR-cas9 technology in *Leishmania* provides opportunities to introduce gene modifications/deletions creating parasite strains lacking specific antigens, a valuable tool to assess the importance of putative virulence factors and to formally distinguish between bystander and antigen-specific immunity following vaccination. In addition, CRISPR-cas9 underpins the development of live GA *Leishmania* vaccines [33].

Whilst immune mechanisms of protection may vary across the spectrum of leishmaniasis, a vaccine with prophylactic efficacy in at least most of the major forms of human disease would be more desirable than one restricted to a single disease entity. Hence, there is considerable value in incorporating antigens shared across parasite species and/or where HLA binding across diverse populations can be demonstrated. Cross-species sequence conservation is a criterion for selection of most subunit vaccines, perhaps exemplified by KMP-11 [37,65], and is obviated in the case of a GA live vaccine. In some cases, sequencing of clinical isolates has been used to fine tune antigens to maximize their chances of immune recognition. For example, the gene encoding hydrophilic acylated surface protein (HASP) B (as used in the ChAd63-KH vaccine) was engineered to produce a synthetic gene product in which the repeat regions retained the diversity and repeat structure found in isolates from across East Africa and India [65].

### 2.4. Appropriate use of preclinical and human models

*Leishmania* can infect and cause disease in a range of mammalian host, including mice, hamsters, guinea pigs, rats, and primates. Numerous reviews have provided in-depth evaluation of some of the advantages as well as the limitations of various models used for the pre-clinical evaluation of vaccine candidates [66,67], and a recent review identified >160 vaccine studies in the literature using such pre-clinical models [68]. Most recently, some of these models, notably in mouse [31] and hamster [69], have been refined by the introduction of sand fly challenge, accommodating the view that vaccine-induced protection may differ between needle inoculation and natural challenge [70]. Whilst model refinement is likely to impact on their predictive efficacy, two limitations remain. First, despite intense study in rodents, understanding of the human response to infection remains somewhat limited, with many clinical studies remaining focused on a handful of cardinal cytokines associated with protective mechanism in leishmaniasis, but which have been largely lacking in predictive power when applied to vaccine studies. A more unbiased evaluation of human immune responses would allow for a broader perspective on the similarities and differences associated with each pre-clinical model. Deep phenotyping of *Leishmania* lesions using bulk or spatially resolved transcriptomics/proteomics have been conducted [71–73] and given the relatively modest changes in clinical protocol or tissue sampling required for such studies, failure to expand this type of research represents a lost opportunity for the field. Analysis costs are of course not insignificant. Second, as well documented for other vaccines-preventable diseases, lessons learnt through clinical trial are essential for effective vaccine selection and development. This iterative cycle is at best rudimentary for leishmaniasis vaccines. There is an urgent need to enhance the throughput of early-stage trials to establish immunogenicity profiles in humans and associate these with outcomes e.g. skin test conversion in a prophylactic setting, or clinical response in a therapeutic setting.

### 2.5. Advancing clinical trials

A search on clinicaltrials.gov (July 2021) using the terms ‘malaria’ and ‘vaccine’ reveals 2 early-phase challenge studies, 171 Phase I studies, 88 Phase II studies and 19 Phase III studies. In contrast, a similar search of ‘leishmaniasis’ and vaccine” yielded 9 Phase I (mostly polyprotein fusions vaccines), 7 Phase II (two Alum-ALM+BCG, three ChAd63-KH and two with recombinant protein vaccines) and 2 phase III (both Alum-ALM+BCG). Only one study (NCT03969134) is currently recruiting. Whilst not suggesting that leishmaniasis vaccine R&D should be on par with that of malaria, these figures do nevertheless illustrate that based on the numbers of candidates tested, the odds are currently stacked against discovering a vaccine for leishmaniasis. Furthermore, the paucity of clinical trials of leishmaniasis vaccines negates iterative learning, of obvious benefit during malaria vaccine development. Failed clinical trials [74], if analyzed in depth, can provide vital clues not only on what constitutes a correlate of protection but also what does not.

The reasons behind the paucity of clinical trials of leishmaniasis vaccines is not for want of candidates, but the combined effects of lack of funding, lack of commercial incentive and clearly defined public health value, poorly predictive models and a reluctance on the part of many researchers to embark on a road so fraught with uncertainty [30,39,75,76]. G-Finder data (https://gfinderdata.policycuresresearch.org/pages/data-visualisations/allNeglectedDiseases) indicates a drop in overall funding for leishmaniasis from 2% of total global R&D spend in 2007–2012 to only 1% from 2013 − 2019 (not including core funding for research organizations involved in leishmaniasis control) with a marked decline in investment from ~60-80 M p.a. in 2007–2010 to almost level funding of $45 M from 2012 onwards ($41 M in 2019). Since 2007, approx. $79 M was earmarked specifically as vaccine research (~12%). In contrast, over the same period drug development received ~ $223 M (33%). Hence, even within funding dedicated to leishmaniasis, vaccine development is apparently not highly prioritized by funders. There may be many factors accounting for this, including proven past successes and the effectiveness of a structured approach to drug development facilitated by DNDi and greater investment in drug discovery infrastructure for neglected diseases e.g. the Tres Cantos Open Lab Foundation (https://www.openlabfoundation.org/AboutTheOpenLab) established by GSK and the Novartis Institute for Tropical Diseases (https://www.novartis.com/our-science/novartis-institutes-biomedical-research/research-locations/novartis-institute-tropical). Furthermore, vaccine development remains a highly risky enterprise with a much lower expected return than drug development, especially for oncology and rare diseases with their high prices. This is even more true for diseases primarily affecting low-income settings. As a result, private funding (in particular venture capital, often critical for the transition into clinical development) is even more rarely directed toward those vaccines.

As discussed below, reaping the benefits of discovery science for vaccine development will require targeted research aimed at building a public health and commercial value proposition to support the investment needed to redress this imbalance. Our own experience has been that ~$2 M was sufficient to develop a new candidate vaccine from conception through to completion of a first-in-human trial. If combined with human infection models [77,78,79] (and Parkash et al., this volume), we estimate that efficacy data on similar new vaccines could be achieved for a total development cost of under $4 M. Relative to the investment in vaccine antigen selection and other aspects of discovery research, this appears a rather modest sum. The leishmaniasis vaccine research development community should raise the bar and set an ambition for at least one new candidate vaccine to enter clinical development every other year. We should take advantage of investments being made in vaccine manufacturing infrastructure for example by UK Research and Innovation [80] and align our plans to benefit from such resources. Should more than one vaccine candidate prove safe and immunogenic in first-in-human trials, well-designed adaptive trials should be employed in controlled human infection models and/or Phase II clinical trials to allow comparability of performance and ease of down-selection.

### 2.6. Modeling the impact of vaccines

Unsurprisingly, the epidemiology of leishmaniasis is complex and varied, particularly so for VL where epidemic cycles are the norm. Mathematical models have played an important role in underpinning strategies for the control of NTDs [81] including the campaign to eliminate VL from the Asian sub-continent [82]. Most research has focused on improving understanding of transmission dynamics for example on a regional scale as for VL in the Indian subcontinent [83], to monitor outbreaks or to assess different transmission modes [84]. More recently, spatio-temporal models have been developed focusing on household or community data [85–87] highlighting the heterogeneity of disease incidence and transmission. In contrast, few studies have used epidemiological modeling to predict how vaccines may serve as effective public health measures. For canine VL, models have been used to illustrate the potential of vaccines to reduce the basic reproductive rate (R_0_) [88,89]. In our recent work [90], we used a set of age-structured deterministic models of VL (Erasmus MC) parameterized with data from Bihar, India [91] to evaluate the degree to which vaccination could provide an additional tool for VL elimination in South Asia. We simulated introduction of vaccines with a variety of different characteristics and found that those which prevented the development of clinical VL, or reduced host infectiousness were likely to have most significant impact. In addition, we found that a vaccine which prevented the development of PKDL would be highly effective at sustaining the VL elimination target once reached through existing control measures, supporting data from spatio-temporal modeling that also suggests PKDL patients play a significant role in VL transmission [92].

Further studies of this type, using spatio-temporal models that allow assessment of the impact of vaccines targeting different aspects of disease natural history, deployed with different schedules and with differing efficacy are urgently needed. In addition, expanding the modeling of other forms of leishmaniasis including zoonotic cutaneous leishmaniasis [93,94] should be seen as a priority, so that the potential global impact of newly developed vaccines might be more fully assessed.

Molecular epidemiology encompassing both parasite and host is also likely to be of increasing importance in designing appropriate early and late phase clinical trials. We currently do not fully understand what dictates variability in clinical cure rates and the potential impact of co-infections, nutritional status or environmental factors on vaccine responsiveness. Biomarkers of disease outcome and treatment response have been developed based on whole-blood transcriptional signatures [95] and similar approaches may provide new tools for monitoring therapeutic vaccine responses. We recently identified a myeloid cell associated gene signature associated with early cure in a small cohort of patients with persistent PKDL in Sudan, though whether this reflects a response to vaccination or provides an insight into the immune status of patients likely to self-cure remains an open question [39]. Parasite genotyping will likely play an important role in clinical trial design, allowing stratification of patients based on parasite genotype and avoiding confounding issues related, for example, to the presence of genetic hybrids.

### 2.7. Identifying vaccine demand

It is commonplace to present leishmaniasis as a disease waiting for a vaccine, built on the argument that vaccination is an achievable goal when seen from an immunological perspective [75]. Whilst this is the case, it represents only half of the argument when considering vaccine development. To fully justify this standpoint also requires an understanding of demand, largely reflecting target population size, schedule of immunizations, roll out approach, longevity of protection and frequency of booster vaccinations. Many of these factors are also reflected in the target product profile (TPP) and have repercussions for choice of vaccine delivery platform, manufacturing approaches and scalability and lead through to the design of early phase clinical studies. Although there have been attempts to formulate a TPP for a visceral leishmaniasis vaccine [96], there have been no substantive attempts to model demand, despite the proven usefulness of this approach in the development of rotavirus and pneumococcal vaccines [97]. This gap is also the result of the absence of an agreed, ideally WHO sanctioned global view, and consequently estimate, of the size of the population at risk of leishmaniasis (both VL and CL). Thus the size of the problem remains unknown.

As a start to addressing this roadblock, we recently developed an in-depth demand forecast for human leishmaniasis vaccines [98]. The approach taken was vaccine-agnostic and examined demand for specific ‘use cases,’ namely prophylaxis targeting either VL, CL or both, and/or therapeutic targeting PKDL. As neither the efficacy nor the required schedule for a vaccine has yet to be established, we modeled a range of potential scenarios each involving roll out initiated with a catch-up campaign but ranging from population coverage in regions at risk with multiple booster vaccinations, to more targeted vaccination campaigns using a more limited vaccination schedule. In each setting, we factored into the simulation the population at risk and their evolving demographics, allowing for a final determination of demand based on the number of doses required. Not surprisingly given the paucity of data and the uncertainty surrounding some of our assumptions, estimates of demand varied under different simulations conditions, from 310 million to 830 million doses required for preventing VL and from 557 million to 1400 million doses required for preventing CL, over a 10-year period (2030–2040). If a stand-alone vaccine was required for targeting PKDL (i.e. vaccines preventing clinical VL did not prevent PKDL), such a vaccine might have more limited demand (~ 330,000 doses over 10 years). These initial results would suggest sufficient demand to support commercial manufacture of prophylactic vaccines targeting CL and/or VL, whereas the limited demand for a vaccine to prevent or treat PKDL might favor philanthropic donors or the use of development pathways favoring orphan indications [98]. Similar considerations regarding limited demand may also come into play when considering the development of vaccines where protection might be confined (e.g. by the nature of the vaccine components) to specific forms of CL, hence targeting only a region-specific portion of the global population at risk. Conversely, should a vaccine be also protective in canids, overall demand estimates (and affordability, see below) might increase, at least for countries where zoonotic VL predominates.

Models of this type can help early-stage vaccine development gain an awareness of the impact of, for example, differing vaccination schedules and to factor this into early-stage vaccine design. However, the future refinement of such models will be dependent on access to more nuanced data on the population at risk for the various leishmaniases, and hence would require significantly improved modeling of disease transmission and the ability to evaluate vaccine impacts when deployed in different ways (e.g. ring vaccination as a means of outbreak prevention).

### 2.8. Understanding the affordability (or ability to pay for) of vaccines

Complementary to an understanding of likely demand for a leishmaniasis vaccine, is an assessment of different countries’ health systems abilities to pay for such vaccines, regardless of whether this is funded entirely through domestic sources or with support from international funders. All countries have limited resources with which to meet the health needs of their populations. It is, therefore, crucial to know whether making funding available for the purchase and deployment of leishmaniasis vaccines would generate a positive or negative impact on the health of the population when compared with other claims upon limited resources. Cost-effectiveness analysis (CEA) can inform this assessment and is widely used in many areas of health care, including for vaccines, such as Human Papilloma virus, Chagas disease, norovirus, and influenza [99,100]. It requires estimation of the health effects of vaccines (for instance, as measured by DALY’s averted), the full commodity and delivery cost net of resource savings generated by avoiding cases of disease. When there is an additional net cost imposed on the health system through acquisition of the vaccine, it is necessary also to know what health gains could have been generated if the equivalent of that net cost was to be invested in other interventions and programs (i.e. the health opportunity cost). This can be expressed as a ‘cost-effectiveness threshold’ reflecting the system’s marginal productivity and hence ability to pay [101].

Studies providing estimates for the cost-effectiveness of leishmaniasis vaccines are few. Lee et al. [102] used a Markov modeling approach to generate an estimate of cost-effectiveness for a vaccine targeting VL in Bihar state, India. Their analysis, based on 2012 data on VL incidence and treatment costs (including for Amphotericin B, hospitalization, loss of earnings etc.), suggested that a vaccine with >50% efficacy would be cost-effective at a vaccination cost (including vaccine components, accessories, storage, distribution, labor, and training) per individual of $350 and one with 25% efficacy would still be cost-effective at $100. However, this study did not take into account the practical considerations of vaccine introduction, such as gradual rollout and wastage. Further, it relied upon very high assessments of how much countries could afford to pay, based upon now discredited former WHO guidance of using a cost-effectiveness threshold equal to a country’s Gross Domestic Product per capita [103] rather than the health system’s ability to pay.

More recently, we have projected the economic feasibility of a leishmaniasis vaccine based on estimates of cost-effectiveness thresholds reflecting health opportunity costs, as well as disease incidence and burden of disease [104]. In contrast to the approach taken by Lee et al., our simulation projects the future value of the vaccine based on a realistic timescale of availability and distribution, accounts for timescales of vaccine rollout and changes in health systems and is an estimate of the full health system cost per vaccinated individual that countries can afford [104]. Given current estimates of population at risk, this analysis suggests that for 13 out of the 24 countries (representing 80% of the global burden of CL and VL) which were analyzed, the projected demand for vaccines between 2030 and 2040 could be afforded at vaccination costs over $3 per course administered under cost-effectiveness considerations, even if the efficacy of the vaccine were as low as 50%. Given the expected manufacturing costs of $2-3, at least for some candidate vaccines with production at scale [105], this implies that the vaccine would be commercially viable, unless the vaccine implementation costs are prohibitive. Furthermore, these calculations may under-estimate significantly the ability to pay due to a number of factors, notably exclusion of vaccine impacts on transmission [106,107], under-reporting of disease incidence [5,108], under-appreciation of disease burden [5] and treatments costs due to HIV co-infection [109].

Capturing a vaccine’s potential economic value as described above will also provide valuable insights spanning the development process and that can be used to inform the development of TPPs, including generating estimates of manufacturing scale and costs, efficacy targets, refining target populations, and target demand.

### 2.9. Increasing advocacy for leishmaniasis vaccines

Numerous examples exist where the R&D and public health and financial communities have come together to facilitate change in the vaccine or drug development landscape through product development partnerships (PDPs) Notable examples include the Medicines for Malaria Venture (https://www.mmv.org), the TB Alliance (https://www.tballiance.org) and the International AIDS Vaccine Initiative (https://www.iavi.org). Unlike these PDPs that focus on a single disease, the Drugs for Neglected Diseases Initiative (DNDi) takes a somewhat broader approach and has been spectacularly successful, delivering eight new treatments for five neglected tropical diseases. For leishmaniasis, DNDi have supported the introduction of two new treatments for VL and currently have seven new chemical entities under development (one preclinical, five in Phase I and one in proof of concept). Likewise, the European Vaccine Initiative (EVI) (https://www.euvaccine.eu) promotes vaccine R&D and supports clinical development of vaccines for malaria, leishmaniasis, Shigella, Nipah, and Zika viruses, as well as more recently COVID-19. In addition, EVI supports cross-cutting platforms providing important resources for the vaccine community, including coordinating access to critical EU-funded infrastructure for clinical trials (TRANSVAC2: https://www.transvac.org/transvac2). Thus, through EVI a framework may already exist to expand advocacy for leishmaniasis vaccine development without the burden of establishing a new entity.

The importance of making meaningful progress toward ‘joining the dots’ across these diverse aspects of the vaccine development process has been similarly articulated by Hotez and colleagues in their broader ‘call for action’ for NTD vaccines [76]. The recent pandemic of SARS-CoV-2 has highlighted how research communities nationally and internationally can be brought together to achieve unprecedented gains in terms of both fundamental knowledge and translational impact. For small research communities already hampered by fragmentation and limited resources, such as those engaged in leishmaniasis vaccine development, this must provide a lesson on how future progress can be made to overcome the roadblocks of the past.

## 3. Expert opinion

Developing new measures to control leishmaniasis remains as important as ever. In drug development, DNDi has helped established a robust pipeline of preclinical candidates and a clear pathway for translational development (https://dndi.org/research-development/portfolio). Whilst the former is also true of vaccine discovery research, the latter is sorely missing. For vaccines against leishmaniasis to become a reality, previous roadblocks need to be overcome, through increasing collaborative working, the development of shared resources and harmonized approaches to preclinical and clinical evaluation and a commitment to the use of innovative cost-effective clinical trials coupled with state-of-the-art approaches to identify potential correlates of vaccine-induced immunity. The vaccine R&D process needs to be supported and informed by the greater use of epidemiological modeling to evaluate the true burden of disease and size of the population at risk, predict the benefits of vaccines in different disease settings and through the application of economic modeling to ensure vaccines being developed are suitable for and will be used by the desired market. On this basis, realistic estimates of demand need to be continuously updated to inform decisions of vaccine developers and ministers of health. These goals are challenging, but by taking the first steps toward developing a shared collective ambition, the research community may generate sufficient leverage to stimulate greater funder awareness of the progress made and the potential public health benefits of vaccination and help secure delivery of the first registered vaccine for human leishmaniasis.

Though prophylactic vaccines may provide maximal public health benefit, the role of therapeutic vaccines, used alone or in combined immune-chemotherapy, should not be ignored and progress here may be facilitated by greater integration of drug and vaccine development.

If successful, over the next five years researchers at the fore of human and veterinary vaccine development will have taken the first steps to developing a collaborative network showcasing developments in the field of leishmaniasis vaccinology and the evidence-base supporting the argument for vaccine manufacture and clinical evaluation as well as for country adoption. They will continue to feed the pipeline of vaccine candidates through discovery research that exploits cutting-edge multi-omics approaches to studying parasite biology and disease pathogenesis in humans and animal models. There will be established means to efficiently test such candidates in validated preclinical models utilizing diverse delivery systems (mRNA, viral vectors, adjuvanted protein), and a critical and robust approach to vaccine down-selection based on an expanded portfolio of human infection models. Funding for leishmaniasis vaccine development will be sought not in a piecemeal manner, where the likelihood of return is low, but through longer and larger awards. These will support a collective interdisciplinary vision that is cognisant of the role that vaccines play in a broader ‘one health’ solution to the challenges posed by leishmaniasis. Within 5 years, safety, immunogenicity and initial efficacy data should have been obtained for at least two vaccines, and up to three new candidates will have moved toward clinical development, as a mark of renewed commitment and ambition.

## Article Highlights

- Identification of correlates of protection and pathogenesis – a key gap in scientific understanding requiring greater in-depth immunological analysis of cohorts, vaccine trials and the use of controlled human infection models
- Understanding the role of parasite genomics in determining (potential) variations in vaccine efficacy - whilst candidate vaccine antigens may be shared across species, other genetic determinants of virulence may impact on expression of resistance mechanisms induced by vaccination
- Impact of host nutritional status / co-infections on vaccine efficacy – a somewhat generic roadblock for the development of vaccines for use in LMICs, where studies of candidate leishmaniasis vaccines may contribute
- Increased need for epidemiological modelling of the impact of vaccines on disease burden in different geographical settings – this may be particularly important for zoonotic leishmaniasis as well as for establishing the full burden of the disease and the size of the at-risk population. The latter also to serves as a base for a solid estimate of vaccine demand.
- Economic modelling as foundation for developing a commercial value proposition - beyond determining the effectiveness of vaccines for leishmaniasis, it is necessary to economically assess how much different countries can pay for the health gains offered. This can demonstrate market size and incentivize continued product development.
- Advocacy – there is an increasing need to raise awareness of the case for a leishmaniasis vaccine, spanning grass roots public engagement through to governmental lobbying. Mechanisms and resources for increased advocacy exist but have been largely untapped by the leishmaniasis vaccine research community.

This box summarizes key points contained in the article.
